# Pre-exposure prophylaxis for transgender women and men who have sex with men: qualitative insights from healthcare providers, community organization–based leadership and end users in coastal Kenya

**DOI:** 10.1093/inthealth/ihab043

**Published:** 2021-07-29

**Authors:** Makobu Kimani, Eduard J Sanders, Oscar Chirro, Nana Mukuria, Shally Mahmoud, Tobias F Rinke de Wit, Susan M Graham, Don Operario, Elise M van der Elst

**Affiliations:** KEMRI-Wellcome Trust Research Program, Kilifi, Kenya; KEMRI-Wellcome Trust Research Program, Kilifi, Kenya; Nuffield Department of Medicine, Oxford University, Oxford, UK; KEMRI-Wellcome Trust Research Program, Kilifi, Kenya; KEMRI-Wellcome Trust Research Program, Kilifi, Kenya; KEMRI-Wellcome Trust Research Program, Kilifi, Kenya; Amsterdam Institute for Global Health and Development, Department of Global Health, University of Amsterdam, Amsterdam, The Netherlands; University of Washington, Seattle, WA, USA; Brown University, Providence, RI, USA; KEMRI-Wellcome Trust Research Program, Kilifi, Kenya

**Keywords:** Kenya, MSM, pre-exposure prophylaxis, transgender women

## Abstract

Transgender women (TW) and men who have sex with men (MSM) in Kenya are disproportionately affected by human immunodeficiency virus (HIV) and would benefit substantially from pre-exposure prophylaxis (PrEP). We conducted focus group discussions (FGDs) with healthcare providers (HCPs) and TW/MSM leadership and in-depth interviews (IDIs) with PrEP-experienced MSM and TW to learn about perceived and actual barriers to PrEP programming. Eleven HCP and 10 TW/MSM leaders participated in FGDs before PrEP roll-out (January 2018) and 12 months later. Nineteen PrEP end-users (11 MSM and 8 TW) participated in IDIs. Topic guides explored PrEP knowledge, HIV acquisition risk, gender identity, motivation for PrEP uptake and adherence and PrEP-dispensing venue preferences. Braun and Clarke thematic analysis was applied. Four themes emerged: limited preparedness of HCPs to provide PrEP to TW and MSM, varied motivation for PrEP uptake and persistence among end users, lack of recognition of TW by HCPs and suggestions for PrEP programming improvement from all stakeholders. Providers’ reluctance to prescribe PrEP to TW and distrust of TW towards providers calls for interventions to improve the capacity of service environments and staff HIV preventive care. Alternative locations for PrEP provision, including community-based sites, may be developed with TW/MSM leaders.

## Introduction

Transgender women (TW) and men who have sex with men (MSM) are among key populations with the highest human immunodeficiency virus (HIV) incidences in sub-Saharan Africa (SSA).^1–3^ A study conducted in coastal Kenya produced one of the first known reports of HIV incidence among TW in SSA, which was estimated at 20.6 (95% confidence interval [CI] 6.6 to 63.8) per 100 person-years.^1^ Two subsequent studies reporting incidence estimates among TW in Nigeria (23.8 per 100 person-years)^4^ and in South Africa (31.0 per 100 person-years)^5^ provided corroborating evidence for the high HIV incidence estimate among TW in SSA. The HIV incidence estimate for TW in coastal Kenya was substantially higher (20.6 per100 person-years) than that of MSM in the same location (5.1 per 100 person-years).^1^ HIV incidence in the general population in Kenya has been reported at 0.14 per 100 person-years (95% CI 0.06 to 0.23).^6^ High HIV incidences for both TW and MSM indicate an unmet HIV prevention need and that both groups would benefit from pre-exposure prophylaxis (PrEP).

Since 2017, Kenya's national antiretroviral therapy programme has offered PrEP at most government hospitals.^7–9^ However, MSM and TW are not specifically targeted for PrEP uptake in Kenya, as eligibility criteria do not specify assessment of anal intercourse or same-sex practices as behavioural risk factors.^9^ In a study among 459 HIV-negative MSM from western Kenya, PrEP awareness was moderate (64.3%) but willingness to use PrEP was relatively low (44.9%).^10^ Individual and interpersonal factors such as self-efficacy, perceived ability to use PrEP and indicators of social support were significantly associated with PrEP awareness and acceptability.^10^ This suggests that targeted educational and psychosocial interventions can increase PrEP uptake.^11^ In coastal Kenya, almost all MSM and TW who were informed about an upcoming PrEP study expressed the desire to take it when it was made available.^1,11^

Two studies conducted in coastal Kenya provide insights into challenges with PrEP adherence among MSM and TW. Among participants enrolled in an observational cohort involving monthly follow-up, HIV incidence among MSM with access to programmatic PrEP was high (3.9 per 100 person-years) and did not differ by self-reported PrEP use. Additionally, only one in seven MSM participants in the study who reported taking PrEP had protective tenofovir concentrations and four of five MSM who acquired HIV reported PrEP use but did not have detectable tenofovir concentrations in their plasma.^12^ In the second study, involving 42 MSM and 11 TW who had started PrEP and were in quarterly follow-up, almost 40% of TW and no MSM had protective tenofovir concentrations measured 6–9 months after PrEP initiation.^13^ Qualitative interviews in the latter study suggested that TW had a more complete understanding of the benefits of PrEP compared with MSM.^13^

There are multiple community-based organizations (CBOs) exclusively dedicated to TW and MSM programming in Kenya, often supported by bilateral or multilateral donors. Most of these CBOs offer HIV prevention services to their members, and some aspire to offer PrEP. Engaging the leadership of these organisations may provide insights on how to improve PrEP programming for TW and MSM in the region, especially as stigma might undermine their interactions with healthcare providers (HCPs).^14^

The aim of the present study was to explore the opinions of both HCP and CBO leaders on challenges and suggestions for improvement to PrEP programming in Kenya. Additionally, the study set out to understand the experiences of MSM and TW PrEP end users. To address this aim, we explored insights from HCPs and TW/MSM CBO leadership on PrEP programming in the area and triangulated these findings with experiences of TW and MSM PrEP end users. We selected these groups as they are intimately linked to either PrEP service provision or had in-depth knowledge and their opinions could influence future PrEP programming success.

## Methods

This qualitative study took place between January 2018 and 2019 in coastal Kenya. Respondents included HCPs from a subcounty hospital, leaders of local MSM/TW-themed CBOs and PrEP-experienced MSM and TW end users participating in a cohort study at the Malindi hospital.^13^ Interviews with PrEP experienced MSM and TW were held in a private space in the hospital with just the interviewer and the respondent. This allowed for open and honest response to probes. Focus group discussions with HCPs were held within the comprehensive care clinic (CCC) in a closed space that HCPs were familiar with. Finally, MSM/TW CBO leaders opted to assemble within their organization offices for the focus group discussions (FGDs). Data collection venues are further described in specific sections below.

### Participants and procedures

#### FGDs

At two time points, i.e. before PrEP availability and 12 months later, we conducted a total of four separate FGDs with 21 participants. Participants were selected based on their role in HIV prevention and care services and PrEP knowledge and experience either as a prescriber or a user. We intended the two data collection time points to help assess for any changes in attitude over time.

Of the two FGDs, the first involved 11 HCPs, who all but one participated at both time points. Maximum variation purposive sampling was used to identify HCP participants. Respondents had to be involved in the provision of HIV treatment or prevention services. HCP respondents included clinicians, nurses, administrators and records officers. The topic guides explored service providers’ recognition of MSM and TW patients, personal values and attitudes towards MSM and TW, perceived impact of PrEP on the local HIV epidemic, risk assessment relevant to PrEP provision and HCP experiences in PrEP provision to MSM and TW.

Similarly we conducted two FGDs with the CBO leaders at time points identical to those with HCPs. Using homogeneous purposive sampling, CBO leaders were interviewed in private offices at an MSM- and TW-themed CBO based in coastal Kenya. FGD participants (n=10) comprised the organizations’ administrators, TW and MSM representatives and TW peer educators. All respondents from the CBO identified as either MSM or TW. Respondents were purposefully selected, as they were strongly connected to the TW and MSM communities in the area. The same respondents participated in FGDs at both time points. The discussion explored PrEP knowledge, understanding HIV acquisition risks, PrEP dispensing venues, perceived and actual challenges experienced by both TW and MSM accessing PrEP and suggestions to improve PrEP programming for TW and MSM in Kenya. Topic guides were developed with input from an expert in MSM- and TW-related research and piloted among a small group of respondents. Discussions were semi-structured and facilitated by MK, while NM co-facilitated, observed and wrote notes. FGDs were conducted in the respondents’ preferred language (Kiswahili) and lasted approximately 90 min.

#### In-depth interviews

Nineteen respondents including 8 TW and 11 MSM with PrEP experience (end-users) were purposively invited for an in-depth interview (IDI). Typical case purposive sampling strategy was used to select respondents who were part of a 12-month PrEP provision cohort.^16^ Briefly, 53 individuals including MSM and TW made quarterly visits to receive PrEP and provide blood samples to assess tenofovir diphosphate levels 6–9 months following PrEP initiation.^13^ The IDI was conducted when respondents returned at the 6-month visit. The interview guide covered PrEP knowledge, motivation for uptake, barriers and facilitators to adherence and perceived support needs to improve PrEP adherence. Additionally, for TW, specific issues such as gender identity, HIV acquisition risk perception, experience with care services by public HCPs and both current or past use of feminizing hormones in regard to PrEP adherence were explored. Interviews were conducted by MK in Kiswahili and lasted for an average of 50 min each.

### Analysis

The FGDs and IDIs were audio recorded and sociodemographic characteristics of each participant obtained. Audio files of the IDIs and FGDs were transcribed verbatim and (where necessary) translated by a qualitative researcher with linguistic competency in Swahili and English. Transcripts were de-identified. Audio files with identifiable data were deleted following transcription. Data were managed using NVivo 11.4 (QSR International, Melbourne, VIC, Australia). Analyses followed Braun and Clarke's thematic approach for qualitative data,^18^ which involved systematic coding, identifying and defining concepts emerging from the data across the data set, mapping the concepts, creating typologies, finding associations between concepts and seeking explanations from the data.

As themes emerged, they were discussed in order of broad to specific, external to internal and macro level to individual level to understand the best strategies for PrEP programming among both MSM and TW communities. Throughout the study process, investigators reflected upon and expressed any assumptions they had about the users’ ability to take up and adhere to PrEP. Similar reflection was made about assumptions of HCP preparedness to prescribe PrEP to MSM or TW. Based on ongoing reflections of their assumptions and positionality, investigators attempted to minimize interpretive bias (i.e. in favour of PrEP) and allow participants’ narratives to guide analysis and interpretations.

## Results

Tables 1 and 2 provide summary characteristics of the study participants (N=40).

**Table 1.** Sociodemographic characteristics of HCPs and MSM/TW community leadership FGD respondents, Malindi, 2018–2019

Study ID	Age (years or range)	Role	Years in HIV work	Gender/behaviour identity or sex
CBO leadership				
CBO/001	26	Program administrator	2	TW
CBO/002	25	Outreach worker	<1	MSM
CBO/003	22	Director	2	MSM
CBO/004	30	Peer educator	<1	MSM
CBO/005	27	Finance officer	<1	MSM
CBO/006	32	Outreach worker	4	MSM
CBO/007	31	Peer educator	10	TW
CBO/008	23	TGNC focal person	4	TW
CBO/009	22	Field worker	2	TW
CBO/010	22	Peer educator	3	TW
HCPs				
HCP/001	>50	Clinician CCC	2	Male
HCP/002	21–30	Records officer	3	Female
HCP/003	31–40	TB clinic community linkage	6	Female
HCP/004	21–30	Records officer	<1	Female
HCP/005	21–30	Data manager	3	Female
HCP/006	41–50	VCT counsellor	7	Female
HCP/007	>50	Nurse	5	Female
HCP/008	21–30	Nurse	2	Male
HCP/009	41–50	HIV services coordinator	15	Male
HCP/010	21–30	Clinician CCC	2	Male
HCP/011	31–40	Clinician CCC	5	Female

TGNC: transgender and gender non-conforming; CCC: comprehensive care centre; TB: tuberculosis; VCT: voluntary counselling and testing.

**Table 2.** Comparison of sociodemographic and sexual risk behaviour characteristics of 19 MSM and TW PrEP end user IDI respondents, Malindi 2018

		MSM (n=11)		TW (n=8)		Total (n=19)	
Characteristic		n	%	n	%	n	%
Age group (years)	18–24	2	18	2	25	4	21
	25–34	9	82	5	62	14	74
	>34	0	0	1	13	1	5
Education	Primary	5	46	4	50	9	47
	Secondary	4	36	3	38	7	37
	Higher	2	18	1	12	1	16
Marital status	Single	9	82	6	75	15	78
	Married	2	18	1	13	3	16
	Separated/divorced	0	0	1	12	1	5
Religion	Christian	4	36	4	50	8	42
	None	3	28	0	0	3	16
	Muslim	4	36	4	50	8	42
Condom use	Always	1	9	1	12	2	11
	Sometimes	10	91	7	88	17	89
	Never	0	0	0	0	0	0
Anal sex role	Insertive	10	91	0	0	10	53
	Receptive	0	0	5	63	5	26
	Versatile	1	9	3	37	4	21
Sex work	Yes	5	45	6	75	11	58
	No	6	55	2	25	8	42
PrEP persistence	On PrEP	8	73	7	88	15	83
	Stopped PrEP	3	27	1	12	4	17

### FGD respondents

Most (70%) of the community leaders were <30 y of age, while 40% of HCPs were 21–30 y of age. Half of the leadership respondents identified as transgender women. A majority of HCPs (72%) had between 1 and 5 y of service delivery experience. Almost all (90%) CBO leaders had between 1 and 5 y in HIV service delivery.

### IDI respondents

A majority (73%) of respondents were between 25 and 34 y. Almost half (45%) had only a primary-level education and three of four (78%) reported being single. Most MSM (91%) reported engaging exclusively in insertive anal intercourse, while none of the TW did so. Inconsistent condom use during anal sex was equally high in MSM (91%) and TW (88%). At the time of the interview, 83% of respondents reported taking PrEP.

#### Qualitative findings

Four thematic areas emerged: PrEP provision preparedness, motivation for uptake and persistence among PrEP end-users, limited recognition of the existence of TW and their risk for HIV infection and recommendations for improvement to PrEP programming for MSM and TW. How these thematic areas influence PrEP uptake and adherence are detailed below.

### Preparedness to provide PrEP to MSM and TW

Initially, at the start of PrEP roll-out, HCPs felt ill-prepared to provide PrEP not just to MSM and TW, but to the general population. They expressed the sentiment that PrEP programming had been implemented too quickly based on directives from the ministry headquarters without consultation with implementing healthcare providers.

*‘While the research may have been done and it showed that PrEP works, we are lacking follow-up systems. How many of those that we started in PrEP are still negative. We do not have things in place. We need to have tools and mechanism for follow-up and how to trace outcomes at intervals. Do we have protocols on how often to retest individuals? I feel like we were not ready for the implementation’*. (Cis male HCP)

This sentiment seemed to have tapered off 1 y into PrEP programming. HCPs were more receptive towards PrEP programming during the follow-up focus groups. However, they admitted that they had adopted a ‘passive’ approach to PrEP delivery, preferring potential users to present themselves at the facility rather than actively screening and counselling high-risk patients about PrEP uptake and adherence. As noted in the above quote, they admitted that this passive approach was ineffective and hampered PrEP roll-out success. Among the reasons for their dampened commitment to PrEP provision was an apparent lack of structural resources and support from hospital management.

*‘…but how about the availability of the guideline because even in this facility, we only have the soft copies. It means we need computers and internet to get them* (guidelines). *So, you are in the (comprehensive care clinic) dealing with HIV, but you don't have hard copy. So, the guidelines may be clear, but it is difficult for someone to understand PrEP management…’*.

### Motivation for PrEP uptake among MSM and TW

Despite structural limitations and challenges in the PrEP provision on the part of HCPs, CBO leaders and end-users noted that the desire among MSM and TW to remain HIV negative strongly motivated the demand for PrEP in this group. The most important driver of PrEP uptake was perceiving oneself as having a high risk for HIV infection.

*‘I wish to remain HIV negative. I know that being a trans is putting me at risk for HIV. So, when I heard that PrEP was available here [hospital], I was among the first to ask for it’*. (TW end user)

However, as currently formulated, both MSM and TW perceived that adherence to daily PrEP would be a challenge and expressed a desire for new formulations that imposed less burden on behaviour and memory to self-administer pills.

*‘…I think it is a nuisance when it comes to the daily dosing. Having to remember when to take PrEP, having to interrupt my fun just to take a drug. That is the nuisance…’.* (TW end user)

Although a minority viewpoint, there was an indication that the protection conferred by PrEP use could lead to increased levels of HIV risk-taking, such as having a greater number of sex partners.

*‘…before I knew about PrEP, I had two partners. When I started using PrEP, I added two more [partners], as I felt protected [by PrEP]. Now I have four partners.* (MSM end user)

### Minimal recognition of TW and acknowledgement of increased risk for HIV infection

HCPs minimized the additional risk for HIV acquisition among TW compared with the general population, and some were unaware of TW as a distinct gender identity. For example, an HCP expressed an opinion shared by others in the group:

‘…*they [TW] are just at the same level as anybody else exposed to HIV…They are not at very a high risk of acquiring HIV…’*. (Cis male HCP)

In contrast, both CBO leaders and end users were clear in their recognition of increased HIV risk among TW. Additionally, both CBO leaders and end users advanced strong sentiments about TW's mistrust and vulnerability to stigma with regard to HCPs, resulting in reluctance among TW to seek PrEP and other HIV services at public hospitals. TW participants who were not open about their gender identity expressed frustration due to pressure to assimilate into mainstream society by concealing their gender.

*‘You'll find that for a trans to come out is tricky. They often try to blend in with the general community and try to please the system that does not even care about them or their health issues’*. (Trans female CBO leader)

### Recommendations to improve PrEP programming and reach

HCPs felt that PrEP provision to MSM and TW would improve through diversified delivery models. They expressed the sentiment that more hospital staff, including nurses and outpatient providers, needed training on PrEP to meet the HIV prevention and medication support needs of the local population. The current system, which relied on front-line clinical providers to implement PrEP, imposed a critical bottleneck in PrEP service delivery.

*‘I think PrEP should have taken a different route like maybe to be dispensed at the outpatient or integrated into public health but not here’*. (Cis female HCP)

‘…*in each department like outpatient, maternity and in the wards, in each department, at least we have one person who is aware about PrEP so that out there they can talk about PrEP…’.* (Cis male HCP)

Integrating PrEP counselling and delivery in the public pharmacy system was also recommended as a strategy to increase PrEP reach and minimize burdens in the clinical care system.

*‘I think they should be made available at pharmacies, so that people who do not want to come to the clinic can still access them’*. (Cis male CBO leader)

Notably, HCP additionally expressed a desire to keep their services applicable for the general community population and called for community collaborations with implementing partners to provide tailored linkages for MSM and TW.

*‘We need to work with organizations that are specialized at working with those populations [MSM and TW]. We can engage at the community level (with the ‘general population’)…, and the Goldstar network [implementing partner] with the KP and their community gatekeepers’.* (Cis female HCP)

To increase PrEP uptake among TW, CBO leaders suggested integration with TW-specific services such as feminizing hormone therapy (FHT). They noted that outreach efforts have been made to address PrEP needs for MSM, but TW have not been included in programs.

*‘There are some specific needs like those hormones, those therapy, those for legal, because it is very expensive by the way the legal change that can be a plus for us. Plenty has been occurring and it is focusing on those MSMs’*. (TW CBO leader)

Finally, when asked what would be a deterrent to PrEP uptake, end users unanimously expressed an aversion to paying for PrEP.

*‘If you ask people to pay for prevention services then they just decide to do without those services. So, if you have PrEP in a chemist on sale, the drugs will expire on the shelf’*. (TW end user)

## Discussion

The overall aim of this article was to understand how to improve PrEP programming for MSM and TW. Our findings identified themes important to consider in further efforts to implement PrEP with MSM, TW and other marginalized populations in Kenya. This is especially important as PrEP is theoretically available to all populations at risk for HIV infection.^7,8^ However, previous work has demonstrated that omission of receptive anal sex from PrEP guidelines may result in reduced targeting of MSM and TW, who are at highest risk for HIV acquisition.^15^ In this study, HCPs did not consider TW to be at increased risk for HIV infection and thus did not prioritize PrEP provision to them. Conceivably, some of the highest-risk populations in Kenya may be systematically excluded at the first step of the PrEP care continuum.

TW in this study expressed frustration about feeling invisible. Globally, TW experience systematic erasure of their existence^16,17^ and are often wrongly categorized as MSM.^18^ Indeed, in this study HCPs neither knew of TW nor recognized any differential in HIV acquisition risk between TW, MSM or the general population. This is a problematic position, as HCPs themselves become a barrier to PrEP provision. A 2014 systematic review identified HCPs as potential hindrances to introducing new drugs—due either to a lack of knowledge or fear of additional workload.^19^ Findings from HCPs in our study supported both reasons, as they felt the PrEP program implementation had been extremely rushed and noted that PrEP programming would needlessly increase their workload at the facility. Their description of adopting a passive approach to PrEP provision aligns with these noted barriers. Specifically, potential PrEP users were expected to present themselves at the facility rather than be identified by HCPs thorough discussion of risk factors and active listening. Additionally, HCPs did not want MSM and TW to access PrEP at the public health facility, instead recommending they be served at other implementing partners’ facilities.

HCPs, CBO leaders and end users all seemed to agree that the public health facility was an inappropriate exclusive venue for PrEP provision, albeit for different reasons. Both end users and CBO leaders expressed uncertainty about the commitment of HCPs to provide PrEP. A recent scoping review on PrEP delivery recommended diversification of PrEP delivery away from the traditional face-to-face interaction at a health facility.^20^ Since the PrEP scale-up in Kenya began, differentiated delivery methods have been considered.^8,21^ PrEP access from community pharmacies has been suggested as an alternative dispensing venue, which should be considered in future research. To better serve TW, HCPs will need sensitivity training, while capacities of TW CBO's should be strengthened.^22^

TW in this study expressed a desire for additional services such as gender-affirming therapy, which currently are not available in public health facilities in Kenya. Previously, TW expressed sentiments prioritizing FHT over other services, including PrEP.^23^ When TW programming guidelines are developed, it may be important to consider integration of such services to increase PrEP uptake and retention in follow-up. Indeed, draft guidelines have alluded to the possibility of the provision of gender-affirming therapy for TW in Kenya.^24^ There is, however, a need to exercise caution on PrEP and FHT. Data from the iFACT trial (NCT03620734) among TW in Thailand demonstrated slightly lowered tenofovir blood levels among TW receiving PrEP and also on FHT,^25^ although studies on drug–drug interactions to date have not shown impacts on PrEP protection levels.

Finally, even as end users expressed strong motivation to take up PrEP, adherence to daily PrEP was cited as challenging. Following the findings of the ANRS IPERGAY trial (NCT01473472), event-driven PrEP has been proposed as a solution to challenges of adherence to a daily regimen.^26^ The World Health Organization has been promoting the use of event-driven PrEP as an alternative to daily PrEP for MSM but not for TW.^27^ Results from the HPTN 083 trial (NCT02720094)^28^ indicated that in both MSM and TW, long-acting injectable cabotegravir was statistically superior to daily oral tenofovir containing PrEP.^29^ Upon regulatory approval, HCPs should be encouraged to offer these alternative PrEP deliveries to end users. However, HCPs need to emphasise the importance of quarterly HIV testing, as those on event-driven PrEP will have fewer interactions with HCPs.

Limitations of this research must be acknowledged. First, qualitative methods produce inherently subjective data that may impose limits on the transferability of the knowledge uncovered. Second, HCP respondents were from a large clinical care centre, where there may be concerns about increased workload that are not expressed in smaller facilities. Third, to get varied opinions, HCP respondents included multiple staff of different ranks in the same space. It is possible that hierarchy and power dynamics within groups could have undermined candid discussions. Fourth, while the study design hoped to capture changes over time in attitude towards PrEP provision, the 1-y period may have been insufficient to detect this. Finally, while we made efforts to interview a diverse group of respondents, policymakers, MSM and TW <18 y of age and HCPs not stationed at the CCC were not included. Similarly, any MSM or TW who did not initiate on PrEP were not included in the interviews. Their reasons for not initiating PrEP would be important to understand. It would be important in subsequent studies to explore their opinions on PrEP programming.

### Conclusions

Despite the availability of PrEP, access continues to be limited for both MSM and TW in Kenya. There is a need to rethink PrEP delivery venues and retrain HCPs to accommodate the needs of both MSM and TW. TW programming guidelines, alternative dispensing venues, adequately prepared HCPs and comprehensive services are urgently required.

## Data Availability

As per the Wellcome Trust policy, all data for this study are freely available from the KEMRI-Wellcome Trust data repository (https://dataverse.harvard.edu/dataverse/kwtrp).
